# A pre-rule for the sequential probability ratio test in a between-item grid multidimensional computerized classification test

**DOI:** 10.3758/s13428-025-02600-x

**Published:** 2025-01-29

**Authors:** Po-Hsien Hu, Ching-Lin Shih, Cheng-Te Chen

**Affiliations:** 1 Institute of Education, National Sun Yat-sen University, Kaohsiung, 804201 Taiwan; 2 Institute of Learning Sciences and Technologies, National Tsing Hua University, Hsinchu, 300044 Taiwan

**Keywords:** Multidimensional computerized classification test, Sequential probability ratio test, Conditional latent trait distribution

## Abstract

The measurement efficiency of a grid multidimensional computerized classification test (grid MCCT), which makes a classification decision per dimension, can be improved by taking the correlations between the dimensions into account in the termination criterion. The higher the correlations, the better the improvement in measurement efficiency. However, a termination criterion utilizing inter-dimensional information (i.e., SPRT-C; Liu et al., 2022) was found to yield lower levels of correct classification rates than not utilizing it (i.e., SPRT-SF; Seitz & Frey, 2013) under the between-item grid MCCT when the cutoff was set at the mean of the latent trait distribution. This study proposes a pre-rule to determine whether the SPRT-SF or SPRT-C should be used during the process of classification test administration. Through a series of simulation studies, the results showed that our proposed method (called P-SPRT) can substantially improve upon the SPRT-C in terms of correct classification rates, while maintaining its high measurement efficiency in terms of test length. This paper concludes with a discussion of the findings and further applications.

## Introduction

The purpose of a unidimensional computerized classification test (CCT; Thompson, 2009a) is to classify examinees into one of two or more mutually exclusive categories along a continuum, such as pass/fail or non-mastery/partial mastery/mastery. Because of the complexity of human traits, the need to categorize examinees into a dichotomous category on several dimensions within one test has drawn an increasing amount of attention. It has also led to the creation of multidimensional versions of classification tests (van Groen et al., 2016). For example, the Minnesota Multiphasic Personality Inventory (MMPI-2; Ben-Porath & Butcher, 1989) contains ten clinical scales and three main validity scales that measure an adult’s personality and psychopathology. After completing the inventory, a set of transformed T-scores and cutoffs are used to indicate the level of clinical symptoms on each of the MMPI-2 scales, which makes the MMPI-2 a multidimensional classification instrument.

For this kind of multidimensional instrument, the required number of items to make an accurate classification decision is expected to be large. As more dimensions are measured, more items are required. Therefore, the response burden for participants is also assumed to increase, which might strengthen participants’ motivation not to complete the instrument, yield low response rates, and lead to poor data quality for studies (Jones, 2014; Rolstad et al., 2011). To shorten the test length required for an accurate classification decision, a CCT adopts two important components: a classification-oriented item selection strategy and a termination criterion procedure. The logic of a unidimensional CCT can be extended to its multidimensional counterpart, which is naturally referred to as a multidimensional CCT (MCCT; van Groen et al., 2016).

Within the framework of an MCCT, there are two types of classification decisions: linear and grid. The first type is making an integrated or overall decision (i.e., pass or fail) according to a common dimension that has been projected from multiple dimensions tapped by the MCCT. This is linear classification (Wang, 2019). The other type is making classification decisions on each of the multiple dimensions, which is called grid classification (Wang, 2019). Grid classification can provide diagnostic information on individual dimensions, which is very useful in achievement tests, where many pass/fail decisions have to be made at the same time. This study focuses on the MCCT that utilizes grid classifications. In addition, each test item of the grid MCCT is assumed to measure one of the target dimensions and is hereafter referred to as a between-item grid MCCT.

The sequential probability ratio test (SPRT; Wald, 1947) has been the most popular termination criterion in unidimensional CCT studies. By conducting a hypothesis test (Thompson, 2009b), it is possible to determine which of the two fixed points is more likely to represent an examinee’s ability. Reckase (1983) also showed that the distance between the two points is primarily related to the required number of items and has only a mild effect on classification accuracy when the guessing parameter is considered in the item response function. The SPRT is the most powerful tool that can minimize test length while controlling for type I and type II error probabilities (Lai, 1997; Nydick, 2014).

Several studies have tried to extend the SPRT termination rule to its multidimensional version and have compared the SPRT with other termination rules in a multidimensional scenario. Seitz and Frey (2013) introduced the SPRT to the between-item grid MCCT and applied it to the MCCT. However, Seitz and Frey’s multidimensional version of the SPRT (hereafter referred to as SPRT-SF) was then reduced to a unidimensional SPRT in their study because the provisional latent trait estimation of the non-target dimension was adopted in the likelihood ratio test. A series of simulation studies showed that the MCCT used as many items as its unidimensional counterparts to achieve the same level of classification accuracy. To include the information induced by the relationships between latent traits into consideration, Liu et al. (2022) incorporated the conditional latent trait distribution, instead of the provisional latent trait estimation, into the likelihood ratio test of the multidimensional SPRT (called SPRT-C in their work) based on Seitz and Frey’s (2013) study. The correlations between latent traits can indeed either benefit classification accuracy or shorten test length through a series of simulation studies. They found that given a classification accuracy comparable to that in the unidimensional counterpart, the MCCT incorporated with the SPRT-C required fewer test items and could save up to 32% of the test length when the two test dimensions were at least moderately correlated.

Though the SPRT-C can reduce the test length adequately when the cutoff points are set at the extremes of latent trait distributions, a non-negligible lower classification accuracy than the SPRT-SF was found when the cutoff points were around the distribution means. The classification accuracies of the SPRT-C and SPRT-SF were 82% and 87%, respectively, while the latent trait dimensions were highly correlated, and the cutoff point was set at the mean of the latent trait distribution (Liu et al., 2022). Such situations are not uncommon. For example, using the neuroticism and extraversion subscales (which are negatively correlated with each other) of the Big Five personality scale, an attempt was made to screen out a group of candidates who were more sensitive and extroverted than the average to receive training for psychological counselors. Using the grid MCCT approach, examinees can be categorized into four groups based on their neuroticism and extraversion levels. This method allows the target group to be distinguished simultaneously from the other three groups. In addition to the generality of the situation, the lower accuracy of the SPRT-C is also of interest, and modifications to the SPRT-C are worth suggesting, retaining its advantage of fewer items while holding comparable classification accuracy, which is the main goal of this study.

This article is organized as follows. The concept and procedures for the SPRT are introduced first. The multidimensional version of the SPRT for the grid MCCT proposed by Seitz and Frey (2013) is then presented. After that, the SPRT-C is described and its shortcomings enunciated, and a new strategy that simultaneously takes advantage of the SPRT-SF and SPRT-C is proposed. A series of simulation studies were implemented to investigate the performance of the newly proposed strategy. Finally, the findings, as well as our suggestions and further applications, are discussed.

## Sequential probability ratio test (SPRT)

The SPRT was used to perform binary classification by testing the following two simple hypotheses (Nydick, 2014):

1 $${H}_{0}:{\theta }_{j}={\theta }_{0}-\delta ={\theta }_{L}$$

2 $${H}_{1}:{\theta }_{j}={\theta }_{0}+\delta ={\theta }_{U},$$

where ${\theta }_{j}$ stands for the latent trait of the *j*th examinee, and ${\theta }_{0}$ is the cutoff point that was set by the researcher or practitioner to distinguish whether ${\theta }_{j}$ is above or below it. To sufficiently differentiate the examinee’s latent trait relative to the cutoff point, the SPRT created an indifference region around the cutoff point with its size equal to $2\delta$, and the upper and lower bounds of the indifference region were defined as ${\theta }_{U}$ and ${\theta }_{L}$, respectively. In the CCT process, after an examinee responds to an item, the above hypotheses will be tested, and one of the three decisions (i.e., the examinee’s latent trait is below ${\theta }_{L}$, above ${\theta }_{U}$, or within the indifference region) will be made. Specifically, the SPRT examines the above hypothesis by calculating the likelihood ratio (*LR*) as follows:

3 $$LR=\frac{L\left({\theta }_{U}|\mathbf{u}\right)}{L\left({\theta }_{L}|\mathbf{u}\right)}=\frac{{\prod }_{i=1}^{k}{P}_{i}{\left({\theta }_{U}\right)}^{{u}_{i}}{\left(1-{P}_{i}\left({\theta }_{U}\right)\right)}^{1-{u}_{i}}}{{\prod }_{i=1}^{k}{P}_{i}{\left({\theta }_{L}\right)}^{{u}_{i}}{\left(1-{P}_{i}\left({\theta }_{L}\right)\right)}^{1-{u}_{i}}},$$

where $\mathbf{u}$ is the item response vector for the *k* administered items, and ${u}_{i}=0$ or 1, indicating an incorrect or correct response to item *i*. By comparing the likelihood assuming the examinee’s latent trait at the lower and upper bounds of the indifference region, the *LR* statistics in Eq. 3 can be obtained. A large *LR* statistic implies that the examinee's latent trait should be above the cutoff point, and a small *LR* statistic implies that the examinee’s latent trait should be below the cutoff point. The CCT will stop proceeding to the next item when one of these two decisions is met. In addition, when the $\delta$ becomes smaller, the values of the numerator and denominator of the *LR* statistic will become closer, so more items are required to make an accurate classification decision (Spray & Reckase, 1996).

To determine when to cease the SPRT procedure, Wald (1947) suggested two criteria for the *LR* statistic, *A* = $\frac{1-\beta }{\alpha }$ and *B* = $\frac{\beta }{1-\alpha }$, where $\alpha$ and $\beta$ represent the nominal type I and type II error rates, respectively. When the *LR* statistic is greater than or equal to *A*, the SPRT rejects the null hypothesis ${H}_{0}$ and classifies the examinee’s latent trait as above the cutoff point. When the *LR* statistic is lower than *B*, the SPRT retains the null hypothesis and classifies the examinee’s latent trait as being below the cutoff point. For conditions when the *LR* statistic ranges between *A* and *B*, no decision should be made, and another item should be selected and administered. However, when the test length reaches the maximum, which is pre-specified by the researchers, a forced classification decision is made by comparing the relative distance from the *LR* statistic to *A* and *B* (Spray & Reckase, 1996).

## Multidimensional version of SPRT by Seitz and Frey (SPRT-SF)

The SPRT was extended to a multidimensional version by Seitz and Frey (2013) and applied to the between-item grid MCCT. According to their procedure, binary classifications on each dimension can be carried out as follows:

4 $${H}_{0}^{\left(d\right)}: {\theta }_{j}^{\left(d\right)}={\theta }_{ }^{\left(d\right)}-\delta = {\theta }_{L}^{\left(d\right)},$$

5 $${H}_{1}^{\left(d\right)}: {\theta }_{j}^{\left(d\right)}={\theta }_{ }^{\left(d\right)}+\delta = {\theta }_{U}^{\left(d\right)},$$

where ${\theta }_{j}^{\left(d\right)}$ denotes the *j*th examinee’s latent trait on the target dimension *d*. An indifference region is defined around the cutoff point on dimension *d*, ${\theta }_{ }^{\left(d\right)}$, with the corresponding lower bound ${\theta }_{L}^{\left(d\right)}$ and upper bound ${\theta }_{U}^{\left(d\right)}$. After collecting item responses on the *k* administered items, the SPRT-SF can test the hypothesis according to the following equation:

6 $${LR}^{\left(d\right)}=\frac{L\left({\theta }_{U}^{\left(d\right)}|\mathbf{u}\right)}{L\left({\theta }_{L}^{\left(d\right)}|\mathbf{u}\right)}=\frac{{\prod }_{i=1}^{k}{P}_{i}{\left({\widehat{\theta }}^{\left(1\right)}, {\widehat{\theta }}^{\left(2\right)},...,{\widehat{\theta }}^{\left(d-1\right)},{\theta }_{U}^{\left(d\right)},{\widehat{\theta }}^{\left(d+1\right)},...\right)}^{{u}_{i}}{\left(1-{P}_{i}\left({\widehat{\theta }}^{\left(1\right)}, {\widehat{\theta }}^{\left(2\right)},...,{\widehat{\theta }}^{\left(d-1\right)},{\theta }_{U}^{\left(d\right)},{\widehat{\theta }}^{\left(d+1\right)},...\right)\right)}^{1-{u}_{i}}}{{\prod }_{i=1}^{k}{P}_{i}{\left({\widehat{\theta }}^{\left(1\right)}, {\widehat{\theta }}^{\left(2\right)},...,{\widehat{\theta }}^{\left(d-1\right)},{\theta }_{L}^{\left(d\right)},{\widehat{\theta }}^{\left(d+1\right)},...\right)}^{{u}_{i}}{\left(1-{P}_{i}\left({\widehat{\theta }}^{\left(1\right)}, {\widehat{\theta }}^{\left(2\right)},...,{\widehat{\theta }}^{\left(d-1\right)},{\theta }_{L}^{\left(d\right)},{\widehat{\theta }}^{\left(d+1\right)},...\right)\right)}^{1-{u}_{i} }},$$

where $\mathbf{u}$ represents a response vector. The likelihood ratio ${LR}^{\left(d\right)}$ is calculated by dividing the likelihood of obtaining $\mathbf{u}$ at the upper bound of the indifference region (${\theta }_{ U}^{\left(d\right)}$) on the target dimension *d* as well as the provisional latent trait estimates (${\widehat{\theta }}^{\left(1\right)}, {\widehat{\theta }}^{\left(2\right)}, \dots$ etc.) on the non-target dimensions obtained at the lower bound (${\uptheta }_{ L}^{\left(d\right)}$) (Seitz & Fray, 2013).

In Eq. 6, the likelihood components that consist of the provisional latent trait estimates in the numerator cancel out the likelihood of the denominator, leaving only one term (i.e., ${\theta }_{ U}^{\left(d\right)}$) for calculating the likelihood. Therefore, Eq. 6 can be further simplified as follows:

7 $${LR}^{\left(d\right)}=\frac{{\prod }_{i=1}^{{k}_{d}}{P}_{i}{\left({\theta }_{ U}^{\left(d\right)}\right)}^{{u}_{i,d}}{\left(1-{P}_{i}\left({\theta }_{ U}^{\left(d\right)}\right)\right)}^{1-{u}_{i,d}}}{{\prod }_{i=1}^{{k}_{d}}{P}_{i}{\left({\theta }_{ L}^{\left(d\right)}\right)}^{{u}_{i,d}}{\left(1-{P}_{i}\left({\theta }_{ L}^{\left(d\right)}\right)\right)}^{1-{u}_{i,d}}},$$

where ${\mathbf{u}}_{d}$ represents item responses to the set of items that load on dimension *d* in which the examinees are being classified. Since the SPRT-SF in Eq. 7 cannot retain any benefit from the between-dimensional correlations, its measurement efficiency is not expected to be better than its unidimensional counterpart. Therefore, the SPRT-SF can be considered to be conducting multiple unidimensional SPRTs sequentially.

## Multidimensional SPRT with a conditional latent trait distribution (SPRT-C)

To improve the measurement efficiency of the SPRT-SF, the auxiliary information provided from the examinee’s latent traits of a non-target dimension can be incorporated into the likelihood of the SPRT. To this end, the expected value of conditional distributions on the other dimensions is introduced to modify the upper and lower bounds of the indifference region on the classifying dimension in the SPRT-C (Liu et al., 2022). To classify examinees on dimension *d*, the *LR* in the SPRT-C is calculated as follows:

8 $${LR}^{\left(d\right)}=\frac{L\left({\theta }_{U}^{\left(d\right)}|\mathbf{u}\right)}{L\left({\theta }_{L}^{\left(d\right)}|\mathbf{u}\right)}=\frac{{\prod }_{i=1}^{k}{P}_{i}{\left({\overline{\theta }}^{\left(1\right)}, {\overline{\theta }}^{\left(2\right)},...,{\overline{\theta }}^{\left(d-1\right)},{\theta }_{U}^{\left(d\right)},{\overline{\theta }}^{\left(d+1\right)},...\right)}^{{u}_{i}}{\left(1-{P}_{i}\left({\overline{\theta }}^{\left(1\right)}, {\overline{\theta }}^{\left(2\right)},...,{\overline{\theta }}^{\left(d-1\right)},{\theta }_{U}^{\left(d\right)},{\overline{\theta }}^{\left(d+1\right)},...\right)\right)}^{1-{u}_{i}}}{{\prod }_{i=1}^{k}{P}_{i}{\left({\stackrel{-}{\theta ^{\prime}}}^{(1)},{\stackrel{-}{\theta ^{\prime}}}^{(2)},...,{\stackrel{-}{\theta ^{\prime}}}^{(d-1)},{\theta }_{L}^{\left(d\right)},{\stackrel{-}{\theta ^{\prime}}}^{(d+1)},...\right)}^{{u}_{i}}{\left(1-{P}_{i}\left({\stackrel{-}{\theta ^{\prime}}}^{(1)},{\stackrel{-}{\theta ^{\prime}}}^{(2)},...,{\stackrel{-}{\theta ^{\prime}}}^{(d-1)},{\theta }_{L}^{\left(d\right)},{\stackrel{-}{\theta ^{\prime}}}^{(d+1)},...\right)\right)}^{1-{u}_{i}}}.$$

In the SPRT-C, the likelihood in the numerator and denominator are derived from both (a) the upper or lower bound of the indifference region on the target dimension *d* (i.e., ${\theta }_{U}^{(d)}$ or ${\theta }_{L}^{(d)}$), and (b) the expected values of the conditional distribution for all of the non-target dimensions while conditioning on the ${\theta }_{U}^{(d)}$ or ${\theta }_{L}^{(d)}$ (i.e., $\overline{\theta }$ or $\stackrel{-}{\theta {\prime}}$). By doing this, the between-dimension correlation can be considered during the likelihood ratio calculation, while assuming that the measured dimensions follow a multivariate normal distribution where the mean vector and variance–covariance matrix are known. Take a bivariate normal distribution, for example. The conditional distribution of ${\theta }_{1}$ conditioning on ${\theta }_{2}$ can be obtained from the following equation (Jensen et al., 2000):

9 $$[{\theta }_{1}|{\theta }_{2}=a]\sim N\left({\mu }_{1}+\frac{{\sigma }_{1}}{{\sigma }_{2}}\rho \left(a-{\mu }_{2}\right),\left(1-{\rho }^{2}\right){\sigma }_{1}^{2}\right),$$

where ${\mu }_{1}, {\sigma }_{1}$ and ${\mu }_{2}, {\sigma }_{2}$ are the mean and standard deviation for dimensions 1 and 2, respectively, and $\rho$ is the correlation coefficient between the two dimensions. When classifying examinees on dimension 2 and a cutoff setting at 2 with $\updelta =0.2,$ while assuming $$\left[\begin{array}{c}{\theta }_{1}\\ {\theta }_{2}\end{array}\right]$$ follows a bivariate normal distribution with a mean vector $$\left[\begin{array}{c}0\\ 0\end{array}\right]$$ and a covariance matrix $$\left[\begin{array}{cc}1& 0.8\\ 0.8& 1\end{array}\right]$$, the conditional distribution of ${\theta }_{1}$ conditioning on ${\theta }_{2}$= upper bound is $\left[{\theta }_{1}\left|{\theta }_{2}=2.2\right.\right]\sim N(1.76, 0.36)$, or $\left[{\theta }_{1}\left|{\theta }_{2}=1.8\right.\right]\sim N(1.44, 0.36)$ for ${\theta }_{2}$= lower bound. The *LR* can be calculated as follows:

10 $${LR}^{(d=2)}=\frac{{\prod }_{i=1}^{k}{P}_{i}{\left({\overline{\theta }}^{(1)}=1.76, {\theta }_{U}^{\left(d=2\right)}=2.2\right)}^{{u}_{i}}{\left(1-{P}_{i}\left({\overline{\theta }}^{(1)}=1.76, {\theta }_{U}^{\left(d=2\right)}=2.2\right)\right)}^{1-{u}_{i}}}{{\prod }_{i=1}^{k}{P}_{i}{\left({\stackrel{-}{\theta ^{\prime}}}^{(1)}=1.44, {\theta }_{L}^{\left(d=2\right)}=1.8\right)}^{{u}_{i}}{\left(1-{P}_{i}\left({\stackrel{-}{\theta {\prime}}}^{(1)}=1.44, {\theta }_{L}^{\left(d=2\right)}=1.8\right)\right)}^{1-{u}_{i}}}.$$

**Fig. 1:**
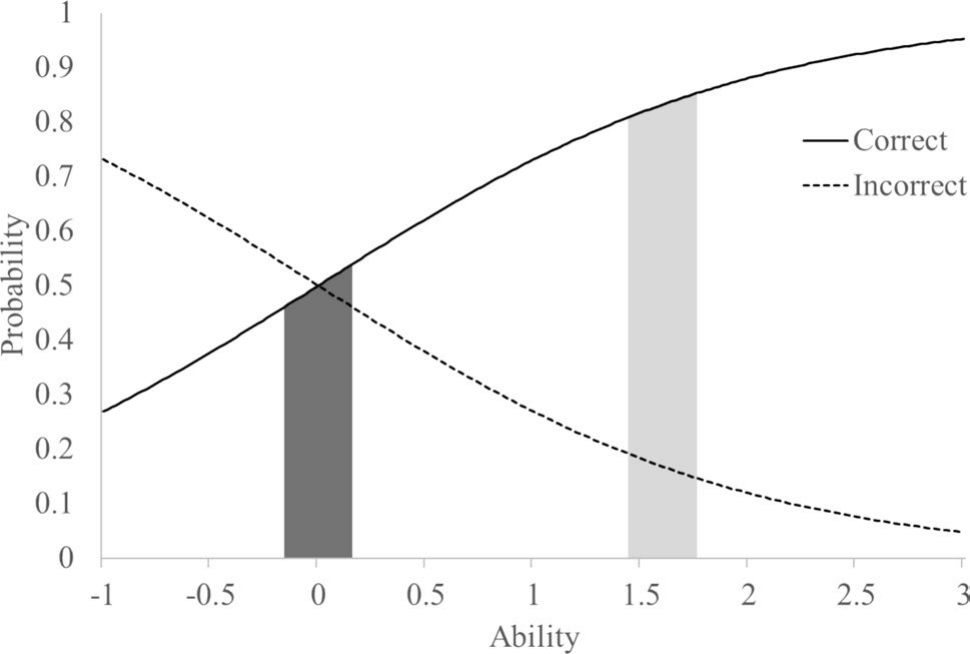
The item characteristic curve of the Rasch model with a difficulty level of 0. *Note*: The light gray area represents the upper and lower bounds of the conditional mean of the non-target dimension when the target dimension's cutoff point is 2, and the dark gray area represents those when the target dimension's cutoff point is 0

When the dimensions are positively correlated, correctly answering items on the non-target dimensions will increase the LR of the target dimension when imputing conditional means to the non-target dimensions. Conversely, incorrect answers will decrease the LR. Building on the previous example, suppose the cutoff point for the non-target dimension is 0. According to the SPRT and Rasch model (1960), items with difficulty levels close to 0 are selected for testing. The probabilities of correct (solid line) and incorrect (dashed line) responses are illustrated in Fig. 1. The light gray area in the figure represents the upper and lower bounds (1.76 and 1.44, respectively) of the conditional mean of the non-target dimension when the target dimension's cutoff point is 2. If this non-target item is answered correctly, the probability of a correct response (solid line) at the upper bound exceeds that at the lower bound, thereby increasing the LR. This increase in LR enhances the likelihood that the ability of the target dimension is above the cutoff point while simultaneously reducing the likelihood that it is below the cutoff point. Conversely, if the item is answered incorrectly, the probability of an incorrect response (dashed line) at the upper bound is lower than at the lower bound, resulting in a decrease in the LR and increasing the likelihood that the target dimension falls below the cutoff point.

In addition, the dark gray area in Fig. 1 represents the upper and lower bounds (0.16 and − 0.16, respectively) of the conditional mean of the non-target dimension when the cutoff point for the target dimension is 0. The degree of LR change is greater in the dark gray area than in the light gray area, indicating that as the cutoff points of the dimensions become closer, the magnitude of LR change increases. In summary, a positive dimensional correlation will promote concordant classifications and hinder discordant ones, and this effect is influenced by the magnitude of the correlation and the distance between cutoff points.

## Pre-rule for applying multidimensional SPRT with a conditional latent trait distribution (P-SPRT)

Liu et al. (2022) found that the SPRT-C could provide a comparable percentage of correct classification (PCC) with a shorter average test length (ATL) in the MCCT than the SPRT-SF. However, for extreme cutoffs of a two-dimensional CCT, such as (− 3, − 3) or (3, 3), the SPRT-C yielded a similar PCC as the SPRT-SF, with only about 70% of the test length given two highly correlated dimensions ($\rho =.8$). However, the PCC might be compromised to some extent at a specific cutoff point. When the cutoff was at the mean of the examinees’ latent trait distribution (e.g., (0, 0) for the two-dimensional condition), the SPRT-C yielded lower PCC than the SPRT-SF as the dimension correlation increased. For example, when the correlation between the two dimensions was 0.8, the PCC (82.08%) yielded from the SPRT-C was lower than the PCC from the SPRT-SF (87.14%) by about 5% (see Table 1 in Liu et al., 2022). Further examination of misclassifications revealed that the SPRT-C yielded more for a certain type of examinee than the SPRT-SF. Specifically, assuming that latent traits follow a bivariate normal distribution with a correlation coefficient of 0.8 and cutoff points of (0, 0), which equals the mean of the latent traits, the true latent traits of most misclassified examinees by the SPRT-C are located in the second or fourth quadrant on the Cartesian coordinate plane. This is because, when an examinee’s true ability is located at the top left or bottom right of the cutoff point (i.e., one latent trait should be classified as above and the other as below the cutoff point), the correlational information induced by the SPRT-C will mislead the likelihood ratio in Eq. 8 and result in more misclassifications. Because in most conditions the SPRT-C requires fewer items to reach classification decisions than the SPRT-SF, Liu et al. (2022) concluded that the SPRT-C can yield a shorter ATL, yet the SPRT-SF can yield PCCs comparable to or higher than those of the SPRT-C. How the compromise in PCC can be ameliorated for the SPRT-C is of great interest and warrants further investigation.

To synthetically integrate the advantages of the two termination criteria (the high PCC of the SPRT-SF and the short ATL of the SPRT-C), a new strategy is proposed in the current study. By choosing between the SPRT-SF and SPRT-C for their corresponding most-fit conditions, a pre-rule is proposed in this study, hereafter referred to as P-SPRT. Taking a two-dimensional case, for example, to determine the choice of the termination criteria between the two SPRTs, a parameter $\varphi$ was used in the P-SPRT as follows:

11 $$\text{P}-\text{SPRT}=\left\{\begin{array}{c}\text{SPRT}-\text{C}\, for\, \varphi>0 \\ \text{SPRT}-\text{SF}\, elsewhere\end{array}\right.,$$

in which

12 $$\varphi =\rho *\left[\left({\widehat{\theta }}^{\left(1\right)}-{\theta }_{ }^{\left(1\right)})*({\widehat{\theta }}^{\left(2\right)}-{\theta }_{ }^{\left(2\right)}\right)\right],$$

where the parameter $\varphi$ equals the product of the correlation coefficient ($\rho$) of the two dimensions and the differences between the examinee’s latent trait estimates (${\widehat{\theta }}^{\left(1\right)}$, ${\widehat{\theta }}^{\left(2\right)}$) and the cutoff points (${\theta }_{ }^{\left(1\right)}$, ${\theta }_{ }^{\left(2\right)}$) on both the *x*- and *y*-axis. When the product value $\varphi$ is larger than zero, this means that the estimated latent traits relative to the cutoff point are in the same direction as the correlation coefficient, so the P-SPRT would implement the SPRT-C procedure to incorporate beneficial between-dimensional information into the *LR* calculation. In contrast, when the $\varphi$ is negative or equals zero, the relative location of the latent trait estimate and the correlation coefficient are not in the same direction, which in turn leads to the implementation of the SPRT-SF procedure, as the calculation of the *LR* will be adversely affected by the between-dimensional information. In general, the P-SPRT is a mechanism that is used to switch the termination criterion from the SPRT-SF to SPRT-C, or vice versa, depending on the correlation between the two dimensions and the relative position of latent trait estimates to the cutoff point. This mechanism would be applied after every item response until classification decisions were made on each dimension. To make classification decisions in a between-item grid MCCT with the P-SPRT, the following procedure can be followed:

1. Set the cutoff point for each dimension and define the width of the indifference region $\delta$.
2. Estimate the latent trait vectors based on the collected response vector and the maximum likelihood estimation (MLE).
3. Compute the value of $\varphi$ according to Eq. 12 and determine an appropriate termination criterion with Eq. 11 in real time.
4. Make a classification decision by comparing ${LR}^{(d)}$ derived from the selected termination criterion to the predefined thresholds *A* and *B*.
5. Repeat step 2 to step 4 after each item response is collected until classification decisions on all dimensions are made or the maximum test length is reached. As the maximum test length is reached, a forced classification decision can be made by subtracting $\left|\text{log}\,{LR}^{(d)}-\text{log}\,B\right|$ from $\left|\text{log}\,{LR}^{(d)}-\text{log}\,A\right|$. A positive or negative value indicates rejecting or retaining ${H}_{0}$, respectively.

To investigate the performance of P-SPRT in making classifications with the between-item grid MCCT, a series of simulations were implemented as described below.

## Simulation study

### Design

Five thousand simulated examinees with their ability vectors were randomly generated from a bivariate standardized normal distribution with a manipulated correlation. A between-item multidimensional item bank with 300 items measuring each dimension was constructed following a three-parameter model (3PLM; Birnbaum, 1968). The discrimination parameters were drawn from a normal distribution *N*(1,${0.25}^{2}$). The difficulty parameters followed a uniform distribution, *U*(–3.6, 3.6), and the guessing parameters followed a uniform distribution, *U*(0, 0.3). The 3PLM probability was compared with a value randomly generated from *U*(0, 1) to obtain dichotomous item responses. The present study fixed the width of the indifference region at 0.2 around the cutoff point and set both *α* and *β* at 0.05. The maximum test length for each measured dimension was specified as 30, and an examinee was administered at least three items for each dimension to meet the minimum requirement for content validity.

#### Between-dimensional correlation

Four levels of between-dimensional correlation were manipulated to demonstrate various kinds of correlational relationships: 0 represented no correlation, 0.3 represented a weak correlation, 0.5 meant a median effect, and 0.8 showed a strong relationship. One of the motivations for applying the MCCT rather than a unidimensional CCT is the expectation of improving the correctness of the classification and accelerating the classification procedure by utilizing the correlation between the measured dimensions. A comparison of the SPRT-SF and SPRT-C, which was performed by Liu et al. (2022), has already shown that the SPRT-C was not only able to shorten test length, but was also able to save more items when the dimensional correlation became higher. However, as the dimensional correlation increased, the PCC of the SPRT-C was more compromised. Because the P-SPRT was proposed to take advantage of both termination criteria in considering the relative position from the estimated abilities to the cutoff points, it is expected that the P-SPRT will perform better on the PCC than on the SPRT-C under the conditions in which the cutoff point is set at the mean of the population and the two dimensions are correlated. In addition, it was also expected to yield a shorter ATL than the SPRT-SF, as the two dimensions were correlated.

#### Cutoff points

Based on the mean abilities (0, 0), nine sets of equally distributed cutoff points were assumed so that the effect of cutoff points could be sufficiently explored and the results could be generalized to other cutoff points. The cutoff points were (0, 0), (1, − 1), (1, 0), (1, 1), (2, − 2), (2, − 1), (2, 0), (2, 1), and (2, 2). It was expected that the P-SPRT would be able to avoid the lower PCC from the SPRT-C by instantly switching the termination criterion to the SPRT-SF when the cutoffs were (0, 0) or (1, 1). As for the other cutoff points, the performance of the P-SPRT and SPRT-C on the PCC and ATL was expected to be similar.

#### Item selection

In this study, the Bayesian item selection method proposed by Segall (1996) was used for item selection. For all candidate items, the item information is represented by a 2-by-2 information matrix, which incorporates item parameters and the probability of answering the item correctly when abilities are set to cutoff points. In the CCT scenario, selecting items based on the highest item information at the cutoff point, rather than at the estimated ability point, can save approximately 16.7% of items while maintaining equivalent classification accuracy (Spray & Reckase, 1994). The candidate item with the highest determinant of information matrix at the cutoff point is selected and administered, which results in the order of item alternating, but not necessarily, between dimensions tested.

#### Termination criteria

The termination criterion is used to determine whether a test should stop, which strongly influences the measurement efficiency of a CCT. In addition, this paper proposes a new termination criterion, P-SPRT, and its performance relative to SPRT-SF and SPRT-C is of interest. Therefore, this variable was included in the study.

To evaluate the measurement efficiency of the three termination criteria, several dependent variables were used. The most frequently used criteria in the literature were the PCC and ATL. The PCC was calculated as the average percentage of correct classifications for all examinees. When calculating the PCC, the examinee would be deemed a “correct classification” only when they were correctly classified on both dimensions. The ATL was calculated as the average number of items required for a classification decision across the examinees. A derivative indicator of the PCC per item was calculated to compare the contribution of the termination criteria to the PCC at the item level, in which the PCC represented the contribution at the test level. In addition, the LOSS function (Huebner & Fina, 2015; Vos, 2000), which takes the PCC and ATL into account as a comprehensive index, was included as follows:

13 $$\text{LOSS }= 100*{1}_{w}+L,$$

where ${1}_{w}$ is a binary indicator variable that takes a value of 1 or 0 when the examinee is classified incorrectly or correctly, respectively, and *L* is the number of items that were administered to the examinee. The constant 100 is the penalty for an incorrect classification. Lower classification accuracy and a long test length result in high LOSS values. Therefore, a criterion with a lower LOSS value indicates better performance in classification accuracy and measurement efficiency.

The present simulation study, including both data generation and analysis, was conducted using MATLAB R2023b. The resulting figures were then created using the “ggplot2” package (Wickham, 2016) in R.

## Results

The results of the PCC, ATL, PCC per item, and LOSS for all three termination criteria under the manipulated conditions are presented (from left to right, respectively) in Table 1. Here, the results are divided into four groups of conditions in which the two dimensions are uncorrelated or are weakly, moderately, or strongly correlated. In each group, all nine cutoff points are included, and the results are listed.

**Table 1:** Percentage of correct classification (PCC), average test length (ATL), PCC per item, and LOSS function under various conditions (δ = 0.2)

Correlation	Cutoff point	PCC			ATL			PCC per item			LOSS		
		SPRT-SF	SPRT-C	P-SPRT	SPRT-SF	SPRT-C	P-SPRT	SPRT-SF	SPRT-C	P-SPRT	SPRT-SF	SPRT-C	P-SPRT
0.0	(0, 0)	86.00	86.00	86.00	33.65	33.65	33.65	2.56	2.56	2.56	47.65	47.65	47.65
0.0	(1, − 1)	90.96	90.96	90.96	31.52	31.52	31.52	2.89	2.89	2.89	40.56	40.56	40.56
0.0	(1, 0)	88.18	88.18	88.18	32.23	32.23	32.23	2.74	2.74	2.74	44.05	44.05	44.05
0.0	(1, 1)	91.20	91.20	91.20	30.30	30.30	30.30	3.01	3.01	3.01	39.10	39.10	39.10
0.0	(2, − 2)	98.06	98.06	98.06	22.11	22.11	22.11	4.43	4.43	4.43	24.05	24.05	24.05
0.0	(2, − 1)	94.54	94.54	94.54	26.92	26.92	26.92	3.51	3.51	3.51	32.38	32.38	32.38
0.0	(2, 0)	91.70	91.70	91.70	27.63	27.63	27.63	3.32	3.32	3.32	35.93	35.93	35.93
0.0	(2, 1)	94.76	94.76	94.76	25.69	25.69	25.69	3.69	3.69	3.69	30.93	30.93	30.93
0.0	(2, 2)	97.78	97.78	97.78	19.94	19.94	19.94	4.90	4.90	4.90	22.16	22.16	22.16
0.3	(0, 0)	87.18	86.42	87.18	33.43	32.03	29.84	2.61	2.70	2.92	46.25	45.61	42.66
0.3	(1, − 1)	91.18	90.84	91.12	31.36	29.28	30.17	2.91	3.10	3.02	40.18	38.44	39.05
0.3	(1, 0)	89.40	88.46	89.36	32.01	30.13	29.48	2.79	2.94	3.03	42.61	41.67	40.12
0.3	(1, 1)	91.54	90.92	91.38	30.20	29.80	28.87	3.03	3.05	3.16	38.66	38.88	37.49
0.3	(2, − 2)	97.84	97.86	97.84	22.07	21.81	22.02	4.43	4.49	4.44	24.23	23.95	24.18
0.3	(2, − 1)	94.48	94.58	94.50	26.92	25.97	26.42	3.51	3.64	3.58	32.44	31.39	31.92
0.3	(2, 0)	92.60	92.44	92.60	27.56	26.09	25.85	3.36	3.54	3.58	34.96	33.65	33.25
0.3	(2, 1)	94.62	94.54	94.66	25.76	25.67	25.08	3.67	3.68	3.77	31.14	31.13	30.42
0.3	(2, 2)	97.80	97.78	97.84	20.19	20.55	20.46	4.84	4.76	4.78	22.39	22.77	22.62
0.5	(0, 0)	87.04	83.88	87.02	33.51	30.07	27.25	2.60	2.79	3.19	46.47	46.19	40.23
0.5	(1, − 1)	91.60	91.66	91.38	31.53	28.42	29.94	2.90	3.23	3.05	39.93	36.76	38.56
0.5	(1, 0)	89.62	88.42	89.44	32.06	27.74	27.53	2.80	3.19	3.25	42.44	39.32	38.09
0.5	(1, 1)	91.70	90.48	91.58	30.30	27.92	26.60	3.03	3.24	3.44	38.60	37.44	35.02
0.5	(2, − 2)	97.84	97.82	97.84	22.13	21.67	22.06	4.42	4.51	4.44	24.29	23.85	24.22
0.5	(2, − 1)	94.52	94.44	94.50	26.93	25.50	26.28	3.51	3.70	3.60	32.41	31.06	31.78
0.5	(2, 0)	92.44	92.20	92.30	27.46	24.30	24.66	3.37	3.79	3.74	35.02	32.10	32.36
0.5	(2, 1)	94.54	94.08	94.34	25.70	23.81	23.31	3.68	3.95	4.05	31.16	29.73	28.97
0.5	(2, 2)	98.10	97.78	97.96	20.08	19.73	19.51	4.89	4.96	5.02	21.98	21.95	21.55
0.8	(0, 0)	87.48	81.88	87.72	33.48	24.53	22.70	2.61	3.34	3.86	46.00	42.65	34.98
0.8	(1, − 1)	91.20	91.28	91.20	31.26	27.44	29.55	2.92	3.33	3.09	40.06	36.16	38.35
0.8	(1, 0)	89.46	89.68	89.36	32.16	25.13	26.13	2.78	3.57	3.42	42.70	35.45	36.77
0.8	(1, 1)	92.18	89.60	92.08	30.31	22.04	21.14	3.04	4.07	4.36	38.13	32.44	29.06
0.8	(2, − 2)	97.66	97.66	97.66	22.25	21.70	22.17	4.39	4.50	4.40	24.59	24.04	24.51
0.8	(2, − 1)	94.04	94.10	94.04	26.88	25.12	26.08	3.50	3.75	3.61	32.84	31.02	32.04
0.8	(2, 0)	92.02	92.32	92.00	27.79	23.46	24.60	3.31	3.93	3.74	35.77	31.14	32.60
0.8	(2, 1)	94.62	94.46	94.56	25.93	20.55	20.82	3.65	4.60	4.54	31.31	26.09	26.26
0.8	(2, 2)	98.02	97.54	98.08	20.06	14.66	14.47	4.89	6.65	6.78	22.04	17.12	16.39

SPRT-SF = sequential probability ratio test proposed by Seitz and Frey (2013); SPRT-C = sequential probability ratio test with conditional latent trait distribution; P-SPRT = pre-rule for the SPRT-SF and SPRT-C

### Uncorrelated conditions

When the two dimensions were uncorrelated ($\rho =0.0$), the statistics for the PCC, ATL, and LOSS were the same for the three termination criteria. Since no information from the between-dimensional correlation could be used, the dimensions were classified independently, and the two alternative termination criteria, the SPRT-C and P-SPRT, were reduced to the SPRT-SF. Although the three criteria yielded identical results, the cutoff points still influenced the patterns of the PCC and ATL. First, among the nine cutoff points, the PCC was found to be higher when the cutoffs were set at the extremes, and lower at the mean, of the latent trait distributions. Specifically, high PCCs (ranging from 97.78 to 98.06) were observed for the cutoff points far from the population means, such as (2, − 2) and (2, 2). For cutoffs such as (0, 0) and (1, 0), the PCCs only ranged from 86.00 to 88.18. Second, the patterns of the ATL also correlated with the distance from the cutoff points to the population means. The greater the distance, the shorter the ATL. This finding was in line with those of Liu et al. (2022), Nydick (2014), and Thompson (2009a), and the PCC and ATL were merely a reflection of the probability density of the bivariate normal distribution within the indifference region.

### Correlated conditions

**Fig. 2:**
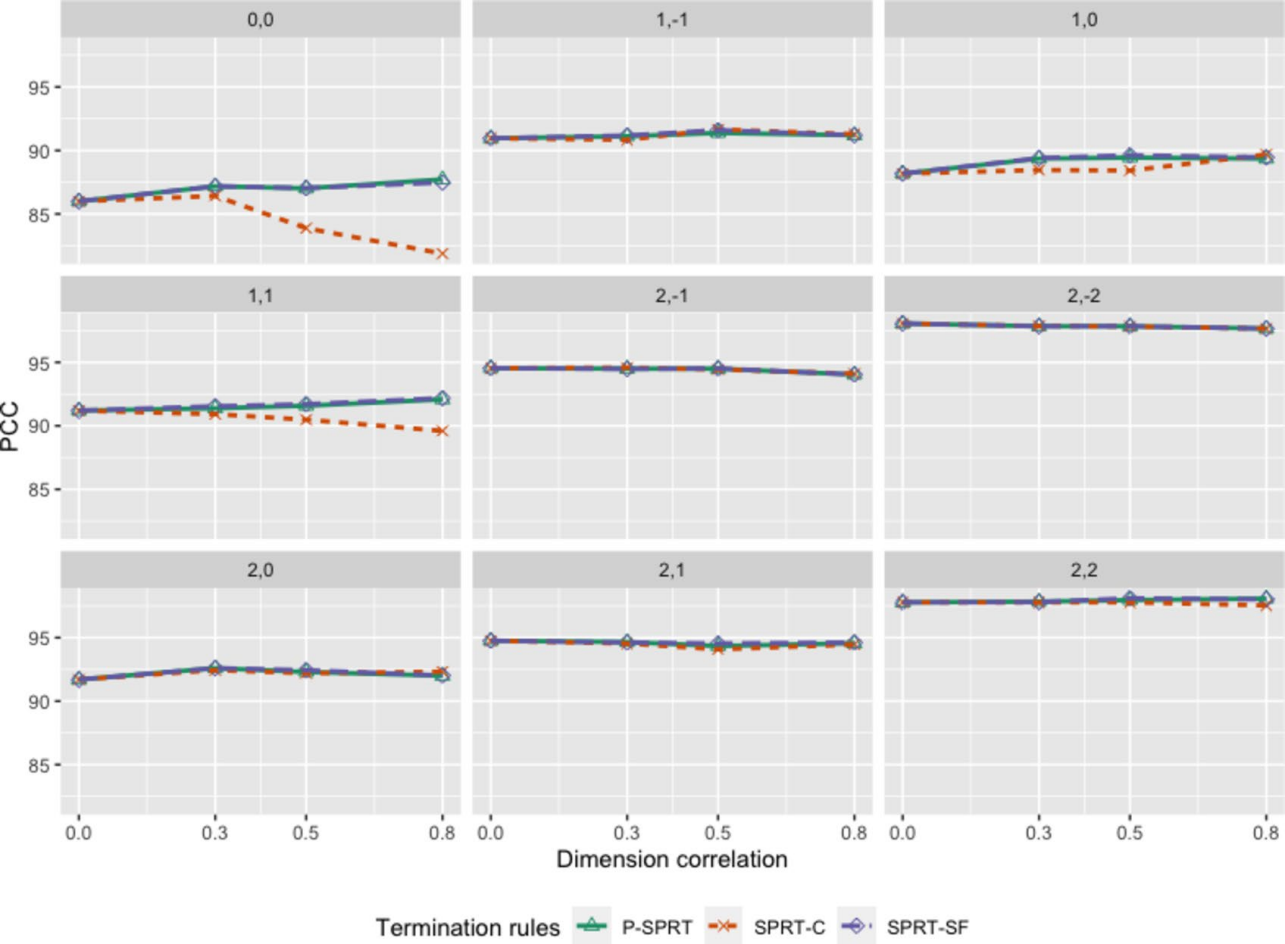
The PCC of termination criteria on different cutoff points and levels of dimension correlation. *Note*: The subtitles represent the location of cutoff points

**Fig. 3:**
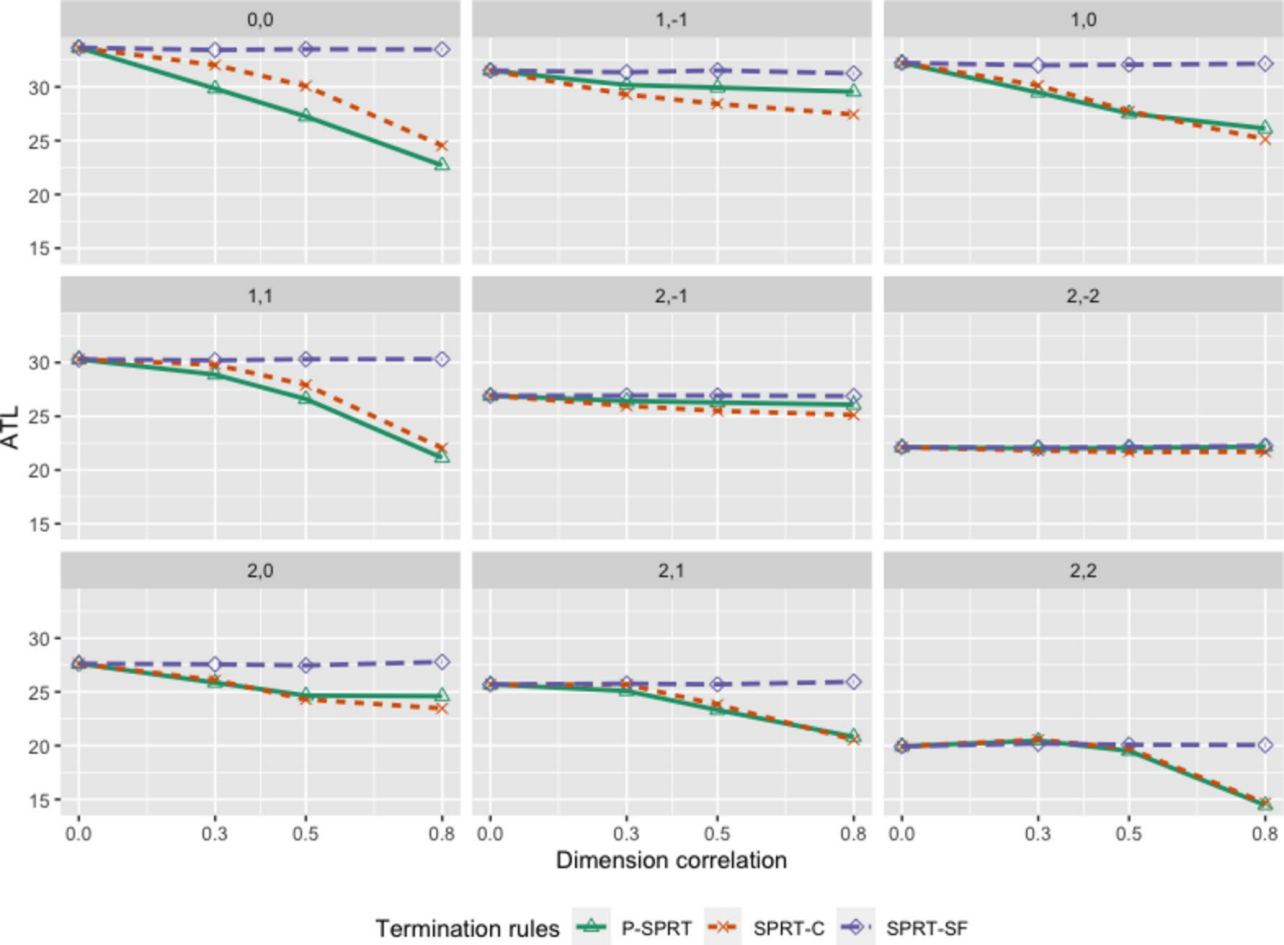
The ATL of termination criteria on different cutoff points and levels of dimension correlation. *Note*: The subtitles represent the location of cutoff points

Figure 2 denotes the relationship of the PCC from the three termination criteria and the dimensional correlation within each cutoff point, whereas Fig. 3 denotes this for the ATL. As the correlation of the two dimensions increased ($\rho =.3$, 0.5, 0.8), the patterns of the PCC and ATL for the three termination strategies became diverse. First, the PCCs of each termination criterion and each dimensional correlation were similar within 7 of 9 cutoff points. However, when the cutoff points were set near the population means, such as (0, 0) and (1, 1), the PCC of the SPRT-C decreased along with the increase in the dimensional correlation, whereas the PCCs of the SPRT-SF and P-SPRT were not affected by the correlation. For example, the PCC of the SPRT-C decreased from 86.42 to 81.88 as the correlation increased from 0.3 to 0.8 when the cutoff points were set as (0, 0). As Liu et al. (2022) found, the PCC of the SPRT-C was compromised when the dimensional correlation was strong and the cutoff points were set near the population means.

Second, unlike the SPRT-SF, the average test length (i.e., ATL) decreased as the dimensional correlation increased, especially under the conditions in which the cutoff points were set on the population means (0, 0) or within the first quadrant, such as (1, 0), (1, 1), (2, 0), (2, 1), and (2,2) from Fig. 3. In contrast, for the conditions in which the cutoff was set on the fourth quadrant, such as (2, − 2), (1, − 1), and (2, − 1), the test length could not benefit very much from the increase in the dimensional correlation, as expected. For example, when the cutoff point = (0, 0), the ATL of the SPRT-C decreased from 33.65 to 24.53 as the correlation increased from 0 to 0.8, whereas the ATL of the P-SPRT also decreased (from 33.65 to 22.70). Third, the P-SPRT outperformed the SPRT-C, as the cutoff points were set at (0, 0) or (1, 1). When the dimensional correlation = 0.8 and the cutoff was set at (0, 0), the ATL, PCC per item, and LOSS of the SPRT-C were 24.53, 2.61, and 42.64, respectively, while the P-SPRT showed better results (ATL = 22.70, PCC per item = 3.86, and LOSS = 34.98). The P-SPRT was not only able to compensate for the drawback of the SPRT-C on the PCC when the dimensional correlation was high but also able to save one to two more items while conducting the MCCT.

Fourth, across all the cutoff points under all the correlated conditions, the values of the PCC per item were 3.46 for the SPRT-SF, 3.81 for the SPRT-C, and 3.85 for the P-SPRT. This demonstrates that a higher PCC could be obtained, as each item was administrated when the SPRT-C or P-SPRT was applied in the MCCT. From the perspective of the average LOSS, the P-SPRT also had the lowest value (31.98) of the three termination criteria, which means that the P-SPRT termination could accelerate the process of the MCCT by using the dimensional correlation without sacrificing the accuracy of the classification. Additionally, when the cutoff was (0, 0) and the correlation was 0.5, the percentage of cases reaching the maximum test length on both dimensions was 4.94% for P-SPRT, 6.64% for SPRT-SF, and 7.32% for SPRT-C. This value not only reflects the efficiency of the MCCT but also implies the potential for classification errors. When examinees are forced to be classified due to reaching the maximum test length, the classification decision is made under insufficient test data, thereby increasing the potential for misclassification. Compared to other termination criteria, the value associated with the P-SPRT is lower, indicating that the P-SPRT exhibits better testing efficiency and is more effective in reducing classification errors. The relationship between test efficiency and classification errors will be discussed later.

## Conclusion and discussion

The SPRT has been used as a termination criterion in CCTs for decades, and it has been found to be useful in classifying examinees into one of two or more mutually exclusive categories (e.g., Spray & Reckase, 1996). In reality, there exists a need for making classification decisions for examinees with shorter test lengths, especially when multiple dimensions are being measured or classified simultaneously. Under that scenario (e.g., the MMPI-2), the grid MCCT can be implemented, but a long test can be expected. Making classification decisions on each dimension has several advantages. First, the examinee’s level of mastery (i.e., mastery, non-mastery, or even partial mastery) on each dimension can be better monitored and then used to provide more information for diagnostic purposes. Moreover, in educational practice, providing more diagnostic information to examinees and teachers, as well as other stakeholders, can lead to more guided instruction in the classroom and more reasonable policymaking for educational authorities. Furthermore, one or more synthetic decisions can be derived from these classifications on each dimension by using methods that have been introduced in the literature (e.g., the composite method). Because the use of multidimensional measurement results in longer tests, finding ways to shorten the test length for the MCCT is certainly of interest and worth further investigation.

With regard to extending the SPRT to its multidimensional version, several studies found it was either not feasible (e.g., Spray et al., 1997) or no more efficient than its unidimensional counterpart (e.g., Seitz & Frey, 2013). With inspiration from the multidimensional computerized adaptive testing, Liu et al. (2022) applied the between-dimensional correlation to the calculation of the likelihood ratio in the SPRT and proposed a new criterion called SPRT-C. Specifically, they applied the conditional distribution to dimensions in which the examinees were not to be classified, so the likelihood ratio for the target dimension could be weighted according to the dimensional correlation. Through a series of simulation studies, they found that the SPRT-C could improve the measurement efficiency of the SPRT-SF on extreme cutoffs, yet SPRT-C yielded lower PCCs than SPRT-SF when the cutoff was set at the mean of the examinees’ latent trait distribution. Though the cutoff was more likely to be set at the extreme of the latent continuum, the preceding finding did represent a drawback for researchers in adopting the SPRT-C as a termination criterion in their studies and needs to be reasonably addressed.

In the current study, a synthetic integration of the SPRT-SF and the more recent SPRT-C was proposed, called the P-SPRT. By switching the SPRT-SF and SPRT-C, as determined by the examinees’ relative position to the cutoff points, the ATL of the P-SPRT was expected to be as short as the SPRT-C, while the level of the PCC remained comparable to that of the SPRT-SF, especially when the cutoff points were set at the distribution means. After a series of simulations, the PCC, ATL, and average LOSS for the three compared termination criteria under various conditions were explored. The results showed that the P-SPRT is capable of maintaining the PCC as the SPRT-SF while keeping the test length short, even shorter than the SPRT-C, under the conditions that the two dimensions are highly correlated and the cutoff points are set close to the population means. In addition, the P-SPRT performed similarly to the SPRT-C with more extreme cutoff points, such as (2, 2). These findings indicated that P-SPRT can reach PCCs comparable to the SPRT-SF with higher measurement efficiency and can be taken into consideration for practical use.

**Fig. 4:**
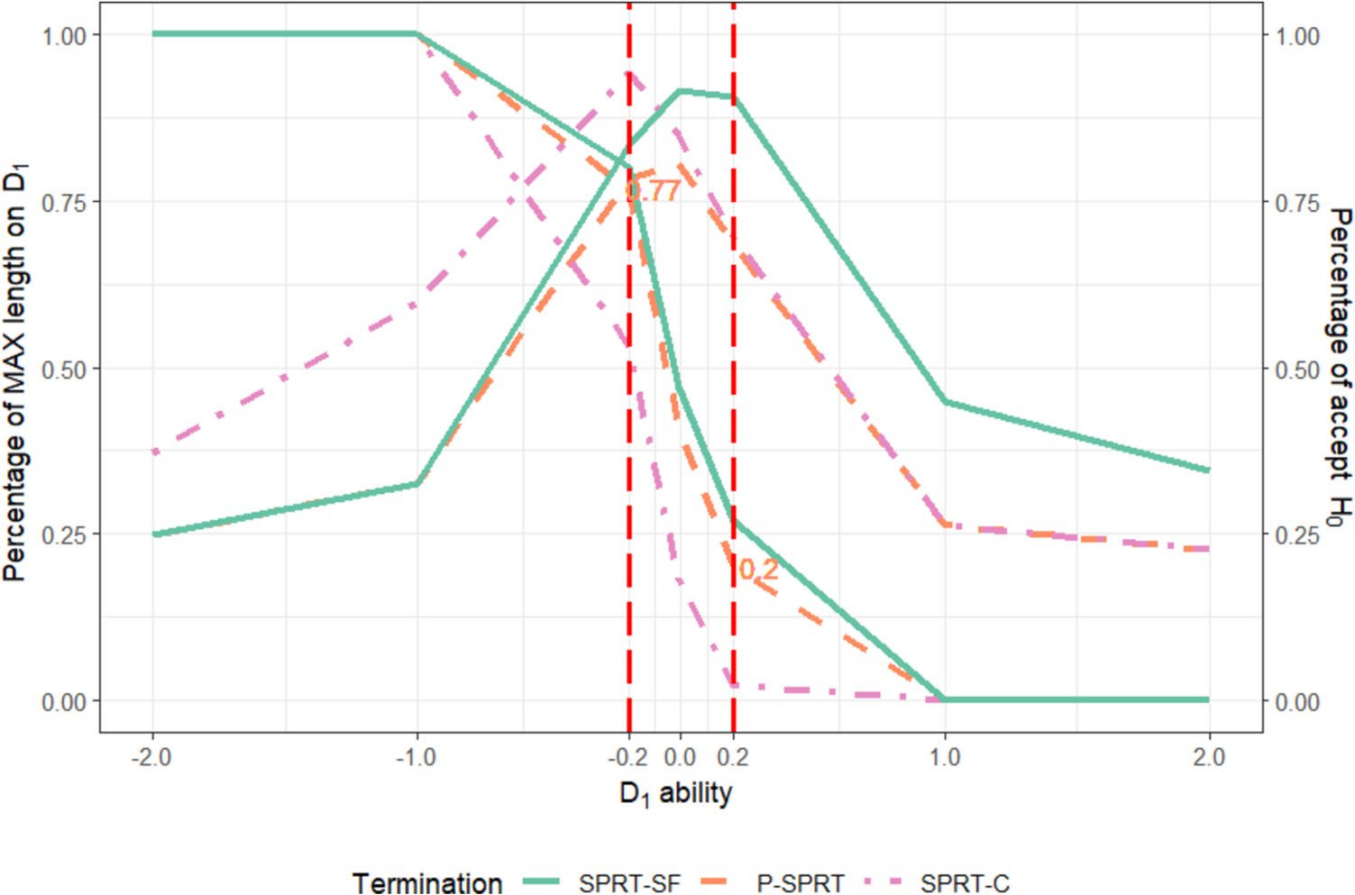
The OC/ATL curves of the compared terminations in the special case simulation. Note: The *x*-axis represents the ability scale of dimension 1. The *y*-axis on the left side of the figure represents the percentage of average test length over the maximum test length (which was set at 30) for dimension 1. The *y*-axis on the right side of the figure represents the OC function. The inverse U-shaped curves represent the ATL pattern, and the ogive curves are OC functions. The two numbers represent the value of the OC function when the ability of dimension 1 is equal to − 0.2 and 0.2

Apart from examining marginal results across examinee abilities, practitioners who are planning an MCCT can further evaluate the accuracy and ATL performance by observing the variation in the percentage of accepting the null hypothesis and ATL as a function of ability—also known as the operating characteristic (OC)/ATL curve (Reckase, 1983)—on one dimension while conditioning the examinee’s ability on the other dimension. This approach also allows for the investigation and comparison of the performance of different termination criteria, particularly when the examinee’s ability is located within the indifference region. Ideally, at the boundaries of the indifference region, the type I and type II error rates should align with the nominal levels (0.05 and 0.05, respectively). To explore this, an additional simulation study was conducted. Most settings were the same as those in the previous simulation study; however, the cutoff point was set at (0,0) and the dimensional correlation for the SPRT-C was set at 0.5 in this context. Examinees’ abilities were set at seven points on dimension 1 (− 2, − 1, − 0.2, −0.01, 0.2, 1, and 2), and the ability of dimension 2 was fixed at 1.00, which is far from the cutoff point. For each pair of abilities, 100 sets of response patterns were generated for the OC. Figure 4 illustrates the percentage of accepting the null hypothesis (i.e., the OC function) and the ATL, in which the ogive curves are OC functions and the inverse U-shaped curve represents the ATL pattern for the three compared termination methods.

The overall pattern of OC and ATL curves for the three termination criteria (i.e., the SPRT-SF, SPRT-C, and P-SPRT) closely aligns with the theoretical OC and ATL functions described by Reckase (1983). The percentage of accepting the null hypothesis decreases as the ability on dimension 1 increases, and the peak ATL occurs near the cutoff point. Like Reckase's (1983) simulation results, the theoretical error rate becomes observable only when a broad indifference region or a large number of items are administered, allowing all examinees to be classified based on the likelihood ratio reaching the predetermined decision points. This limitation arises because some examinees are forced to classify after reaching the maximum test length due to slight variations in the likelihood ratio within a narrow indifference region. Additionally, the steep slope of the OC curve for the P-SPRT, along with its shorter ATL compared to both SPRT-SF and SPRT-C, indicates that the P-SPRT successfully retains the advantages of both methods.

Several issues remain unclear and need further exploration. First, how the P-SPRT performs relative to other termination criteria combined with conditional distribution methods, such as stochastically curtailed SPRT (SC-SPRT; Finkelman, 2008) and the generalized likelihood ratio (Thompson, 2009b), can be further investigated. Second, for examinees whose abilities are near the boundary of the indifference region, the increased error rate caused by forced classification should not be overlooked. Because the type I and type II error rates are both 0.05 in the special case simulation, 95% of examinees with an ability of − 0.2 (i.e., lower bound of the indifference region) on dimension 1 should ideally be classified as below the cutoff, while 5% of examinees with an ability of + 0.2 (i.e., upper bound of the indifference region) may be misclassified. Figure 4 indicates that, with the proposed P-SPRT termination, observed rates of accepting the null hypothesis (i.e., OC) at the lower and upper bounds are 77% and 20%, which are 18% lower and 15% higher than the nominal level, respectively. When the maximum test length increases to 60 items, the OC values are 0.83 and 0.09, respectively. Further increasing the maximum test length could potentially bring the type I and type II error rates closer to their theoretical values; however, this would result in excessively long test lengths, compromising the efficiency and practicality of the assessment. For grid MCCT practitioners, it is recommended that they flag examinees who were classified due to reaching the maximum test length and to closely examine these cases. Further screening methods for these examinees are essential, as relying solely on grid MCCT results for final classification may not be appropriate, especially in high-stakes qualification exams or when test lengths are short.

Third, due to many tests that measure multiple dimensions (≥ 3) simultaneously, the extension of the P-SPRT to higher dimensions needs to be further studied. Fourth, alternative strategies may exist to combine the SPRT-SF and SPRT-C, such as simultaneously applying both stopping criteria and terminating the test when either condition (or both conditions) is met. Finally, application of the P-SPRT to practical test scenarios, such as those in the educational, psychological, and organizational fields, can be implemented to more practically evaluate the performance of the P-SPRT.

## Data Availability

None of the simulation experiments were preregistered. For reproducibility purposes, data files for all simulation experiments and simulation codes are available at https://osf.io/9sbec/?view_only=c8a40e0d905a4f0592ddeaf7121be2ba
